# Acute ischemic stroke during cardiac catheterization: a rare case report in Malaysia

**DOI:** 10.11604/pamj.2024.49.72.45624

**Published:** 2024-11-11

**Authors:** Mei Yin Pong, Jun Fai Yap, Hong Guan Sim, Kee Wei Lee, Anwar Suhaimi

**Affiliations:** 1 Department of Rehabilitation Medicine, Faculty of Medicine, Universiti Malaya, Kuala Lumpur, Malaysia,; 2 Training Management Division, Ministry of Health, Putrajaya, Malaysia,; 3 Institute for Public Health, National Institutes of Health, Shah Alam, Ministry of Health, Selangor, Malaysia,; 4 Department of Social and Preventive Medicine, Faculty of Medicine, Universiti Malaya, Kuala Lumpur, Malaysia,; 5 Department of Biomedical Imaging, Faculty of Medicine, Universiti Malaya, Kuala Lumpur, Malaysia,; 6 Cardiology Unit, Department of Medicine, Universiti Malaya Medical Centre, Kuala Lumpur, Malaysia

**Keywords:** Angioplasty, stroke, percutaneous coronary intervention, case report

## Abstract

Acute stroke occurring during cardiac catheterization is an extremely uncommon procedural complication. There is a significant research gap in the published literature regarding the occurrence of acute ischemic stroke during percutaneous coronary intervention in Asian populations, with most data originating from western countries, underscoring the need for localized studies to develop tailored detection and management strategies. We present the case of a 70-year-old Indian woman with multiple cardiovascular risk factors who experienced an acute ischemic stroke while undergoing percutaneous coronary intervention in Malaysia. We hypothesized that the clinically relevant cerebral infarction might have originated from large plaques located along the aortic arch. Catheterization-related strokes caused by embolization of fresh thrombi forming at the catheter or guidewire tips should be thrombolysed. Choosing the transradial approach reduces stroke risk but requires careful use of appropriately sized instruments and meticulous handling of guidewires to ensure patient safety.

## Introduction

The occurrence of an acute ischemic stroke during coronary angiography is an exceedingly rare and often under-recognized complication. The incidence rate of acute ischemic stroke during coronary angiography ranges from 0.2% to 0.4% and is influenced by procedural factors, such as the complexity of the coronary anatomy and the operator's experience, as well as patient-specific factors like the presence of severe atherosclerosis [1].

The pathogenesis of periprocedural ischemic stroke can involve a variety of embolic sources, including air emboli, atherosclerotic debris, or metallic fragments from fractured guidewires and catheters [2]. The structural composition of the thrombus determines the efficacy of thrombolysis [3]. For example, fresh thrombus newly forming at the catheter or guidewire tips are mostly platelet-rich and thus are responsive to thrombolysis.

Despite the documented risks in the published literature, there is a noticeable research gap regarding the occurrence of acute ischemic stroke during percutaneous coronary intervention (PCI) particularly within Asian populations. Most reported cases originate from western countries, leaving a significant lack of data on how this rare complication, presents and is managed in Asian cohorts. This gap is critical to be addressed, since genetic, environmental, and healthcare delivery issues may adversely impact the outcomes of periprocedural strokes in this region. Addressing this gap at the local level is essential to develop tailored strategies for early detection and subsequent management of this complication in Asian patients undergoing PCI. We report a case of an elderly lady with multiple risk factors of cardiovascular disease who experienced an acute ischemic stroke whilst undergoing PCI in a tertiary hospital in Malaysia. The complexity of managing such a case lies not only in the immediate need to recognize the ischemic stroke but also in balancing the risks of further thromboembolic events with the need for antiplatelet therapy to prevent restenosis of the coronary arteries post-PCI.

## Patient and observation

**Patient information:** a 70-year-old Indian woman with underlying obesity, dyslipidemia, and hypertension was admitted to the cardiology ward following an episode of non-ST segment elevation myocardial infarction. Following the cardiac event, she was compliant with double anti-platelet therapy (Aspirin 100mg once daily, Clopidogrel 75mg once daily), antihypertensive medications (Perindopril 8mg once daily, Bisoprolol 1.25mg once daily), and Atorvastatin 40mg once daily.

The patient underwent cardiac catheterization and coronary angiogram under local anaesthesia via the right radial artery. Despite being asymptomatic, having normal blood pressure readings, and unremarkable pre-procedural blood results, tortuosity was noted at her right subclavian artery during the initial phase of the angiogram, leading to technical difficulties with the guidewire.

**Figure 1:**
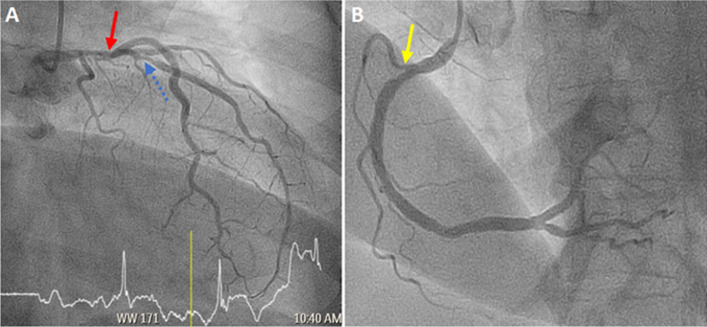
(A, B) coronary angiogram showed left main coronary artery stenosis (red solid arrow), left anterior descending artery stenosis (blue dashed arrow), and right coronary artery stenosis (yellow arrow)

**Clinical findings:** coronary angiogram findings showed right coronary artery stenosis (70%), left main coronary artery stenosis (30 - 40%), and left anterior descending artery stenosis (80 - 90%) (Figure 1). Immediately following the procedure, her Glasgow Coma Scale dropped to E3V2M5. As she was agitated with complaints of acute headache, the angioplasty procedure was abandoned immediately following the acute complaint.

**Figure 2:**
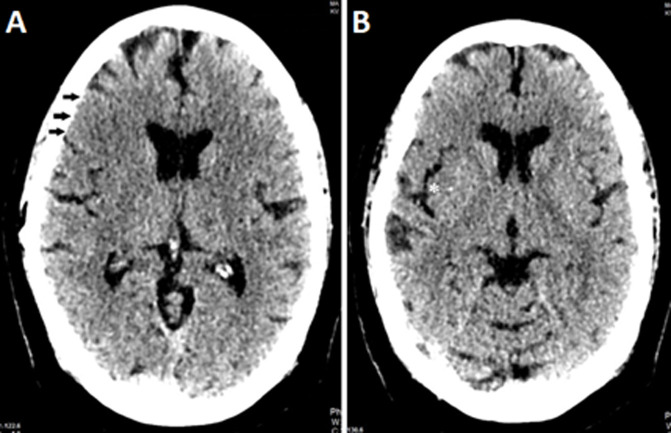
(A, B) brain computed tomography scan showed loss of grey-white matter differentiation (black arrows) and loss of right insular ribbon sign (*)

**Figure 3:**
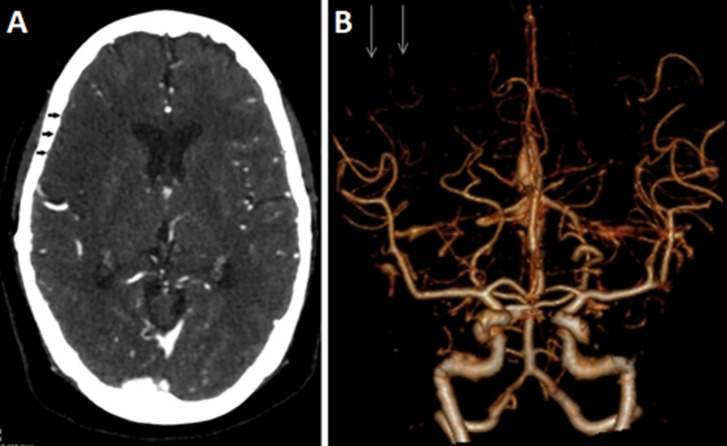
(A, B) cerebral angiography initially showed no contrast opacification at the cortical branches of the right middle cerebral artery (black arrows), but the volume rendering technique showed faint opacification of the cortical branches of the right middle cerebral artery (white arrows)

A stroke alert was initiated. Ill-defined hypodensities with a loss of grey-white matter differentiation were noted on an immediate brain computed tomography scan. These radiological changes involved the right frontal lobe (M1 and M2 territories), and the right insular cortex and were associated with a mass effect, causing effacement of the adjacent cerebral sulci (Figure 2). Loss of the right insular ribbon sign was also noted as one of the early computed tomography findings of acute stroke. A subsequent urgent cerebral angiography revealed an infarction in the right middle cerebral artery (MCA) with an ASPECTS score of 7, along with thrombosis in the distal segment of the right MCA (M4). Volume rendering technique using maximal intensity projection showed faint opacification of the cortical branches of the right MCA (Figure 3). Intravenous thrombolysis with recombinant tissue plasminogen activator (Alteplase) was administered within 4 hours and 18 minutes of stroke onset, resulting in an improvement in her National Institute of Health Stroke Scale from 9 to 6.

**Diagnostic assessment:** she exhibited neurological deficits including left cranial nerve VII palsy (upper motor neuron type), mild dysarthria, reduced strength in her left upper limb [Medical Research Council (MRC) grade 4], and lower limb (MRC grade 4) with a left-sided upgoing Babinski reflex. Cerebellar signs were absent.

**Figure 4:**
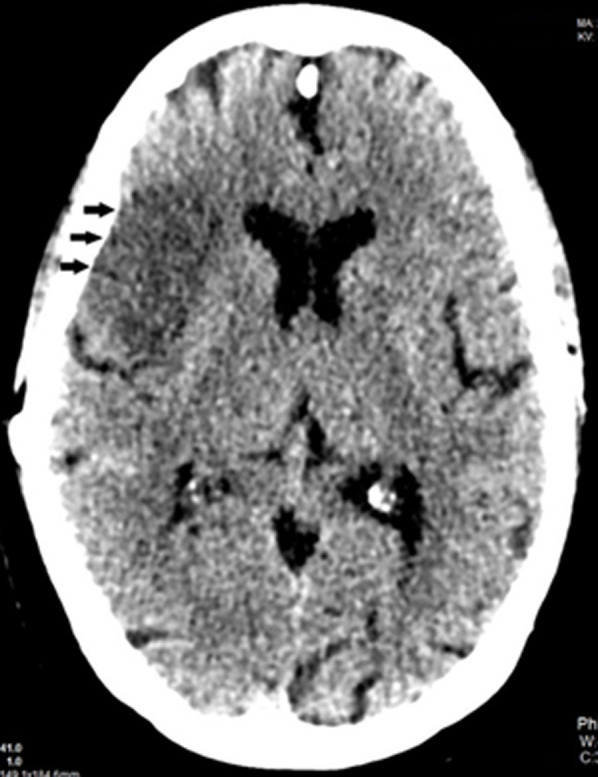
repeated brain computed tomography scan showed right middle cerebral artery territory infarct (black arrows) with no evidence of hemorrhagic transformation

**Diagnosis:** follow-up contrast-enhanced brain computed tomography revealed evolving right MCA territory infarction and old multifocal lacunar infarcts, with no signs of acute intracranial hemorrhage or hemorrhagic transformation (Figure 4).

**Therapeutic interventions:** she had complete bed rest for a week in the ward after the acute ischemic stroke. However, she developed deconditioning. Initially, she was prescribed single antiplatelet therapy during the first week following her stroke. She was then transitioned to dual antiplatelet therapy before being discharged, with a scheduled elective appointment for another PCI.

**Follow-up and outcome of interventions:** upon discharge, the patient was referred to the primary care team for optimization of her traditional cardiovascular disease risk factors, but she eventually missed her scheduled appointments. As a result, her outpatient rehabilitation regimen was not implemented due to her lost-to-follow-up status.

**Patient perspective:** despite the patient's initial compliance with prescribed pharmacotherapies, the sudden onset of stroke symptoms mid-PCI left her feeling vulnerable and anxious about her long-term health outcomes, particularly given the missed follow-up appointments that could hinder her recovery.

**Informed consent:** written informed consent was obtained from the patient included in the study.

## Discussion

Patients with coronary artery disease are at an increased risk of developing periprocedural stroke due to potential atheroma formation in the aorta and aortic arch, which can be dislodged during catheter advancement. Inadequate care whilst advancing the catheter through the aorta may scrap this aortic atheroma off with subsequent embolization to the cerebral arteries. Scraping of aortic plaques frequently occurs during PCI procedures, particularly when larger catheters or intra-aortic balloon pumps are used [4].

Thrombolysis is the preferred treatment for catheterization-related strokes caused by embolization of fresh thrombi forming at the catheter or guidewire tips. The choice of arterial assessment site, catheter size, and careful technique are critical in minimizing stroke risk during PCI procedures. The transradial approach is preferred over the transfemoral route, as the former reduces the risk of cerebral embolization [5]. Transradial approach would completely spare the abdominal and descending thoracic part of the aorta which can be a source of embolic material. Patients with multiple risk factors of atherosclerotic disease, including advanced age, diabetes mellitus, hypertension, chronic kidney disease, or previous cerebrovascular disease are advised to undergo transradial intervention to mitigate the risk of plaque embolization [6-8]. In our case, it was speculated that the clinically relevant cerebral infarction might have originated from large plaques located along the aortic arch. However, confirmative imaging tests such as transesophageal echocardiography and multiscale computerized tomography were not done to search for the atherosclerotic aortic plaques.

While stroke events following PCI have been documented in American patients [9], this case report offers nuanced clinical insights that may be overlooked in broader cohort analyses. Notably Asian populations were at higher risk of acquiring traditional risk factors of cardiovascular disease namely diabetes mellitus or hypertension, which may complicate PCI outcomes [10]. Although the current literature has addressed the overall burden of stroke post-PCI, specific data on subpopulations especially those defined by factors such as ethnicity or comorbidities remain limited. This case report provides an opportunity to highlight these factors, thereby contributing to the growing body of evidence in this domain.

## Conclusion

This case underscores the importance of vigilance and preparedness for rare yet life-threatening complications during a PCI. While the transradial approach lowers the risk of procedure-related stroke, using appropriately sized equipment and practicing catheter manipulation remain paramount.
